# Supplementary material for: Genetic Variability in the Physicochemical Characteristics of Cultivated Coffea canephora Genotypes
Source: Plants (Basel). 2024 Oct 4;13(19):2780. doi: 10.3390/plants13192780 (PMC11479012; doi:10.3390/plants13192780)
Supplement: Supplementary file 1 [file plants-13-02780-s001.zip › plants-3181852-supplementary.pdf]

SUPPLEMENTAL MATERIAL
ATTACHMENT:

Supplementary material. Average grouping, according to Scott Knott at 5% probability, referring to the physicochemical characteristics of the most cultivated genotypes in the Western Amazon, Brazil, evaluated in two measurements, the 2020-2021 and 2021-2022 harvests, part 1.

Genótipos	AE	TA	TTA	pH	TCP	EE
**31-131**	29.87g	4.55i	163.27d	5.12h	15.78d	4.59i
**AR106**	31.13e	4.66h	163.74d	5.07j	14.88e	6.52c
**AS1**	32.86b	4.80f	168.90c	5.13h	15.43d	4.33j
**AS10**	30.09g	4.48j	161.11d	5.23d	15.90d	5.04g
**AS12**	30.16g	4.92e	170.14c	5.20e	14.49f	4.06k
**AS2**	33.21a	4.95e	161.35d	5.24d	14.15g	4.64h
**AS3**	31.22e	4.74g	175.58b	5.17f	15.56d	5.24g
**AS5**	30.51f	4.77f	164.12d	5.20e	14.82e	5.38f
**AS6**	30.58f	4.76f	177.20b	5.19e	14.66e	4.93h
**AS7**	28.97j	4.88e	183.80a	5.17f	15.57d	4.87h
**BAG19**	31.00e	4.67h	148.95g	5.31a	13.74h	4.62h
**BAG21**	28.39l	4.65h	140.23h	5.23d	13.90h	4.71h
**BAG22**	31.60d	4.76f	171.50b	5.15g	15.54d	4.77h
**BAG23**	28.79k	4.91e	149.12g	5.28b	14.36g	4.19j
**BAG24**	29.54h	4.67h	152.42f	5.24d	13.72h	5.57f
**BAG26**	32.16c	5.07c	155.85e	5.20e	16.60c	5.34f
**BAG27**	30.27g	4.92e	163.14d	5.15g	14.3g	4.92h
**BAG28**	30.92e	4.37k	156.92e	5.26c	12.62j	5.16g
**BAG29**	29.46h	4.47j	140.47h	5.29b	15.01e	5.18g
**BAG30**	29.73h	4.18m	151.03f	5.18e	14.54f	4.88h
**BAG32**	31.99c	4.69g	161.18d	5.11i	14.64e	5.24g
**BAG33**	29.11j	4.76f	143.51h	5.32a	14.16g	4.77h
**BAG38**	28.88j	5.07c	173.41b	5.16f	15.54d	5.35f
**BAG41**	28.48l	4.34l	143.10h	5.31a	15.02e	6.56c
**BG180**	29.25i	4.62h	161.41d	5.19e	13.32i	4.57i
**BRS1216**	29.93g	4.84f	165.62d	5.26c	14.58f	5.33f
**BRS2299**	30.04g	4.76f	173.40b	5.14h	16.30c	5.09g
**BRS2314**	29.04j	4.78f	146.62g	5.16f	13.24i	5.60e
**BRS2336**	27.92m	4.30l	154.77f	5.27b	11.63k	5.20g
**BRS2357**	31.01e	5.01d	167.67c	5.15g	18.94a	3.46l

**BRS3137**	29.08j	4.69g	159.27e	5.19e	12.68j	5.60e
**BRS3193**	30.25g	4.79f	179.60a	5.11i	17.42b	5.41f
**BRS3210**	30.51f	4.97d	158.84e	5.23d	14.72e	4.94h
**BRS3213**	28.91j	4.45j	154.33f	5.13h	16.69c	5.70e
**BRS3220**	29.04j	4.72g	157.95e	5.18e	13.81h	5.41f
**CA1**	30.08g	5.29b	184.33a	5.18f	15.03e	6.95b
**GB1**	29.26i	4.74g	159.44e	5.21d	15.61d	5.33f
**GB4**	27.93m	4.83f	156.69e	5.19e	13.42i	5.68e
**GB7**	27.38n	4.40k	157.43e	5.17f	16.24c	5.82e
**GJ20**	30.02g	4.72g	154.16f	5.13h	13.20i	5.82e
**GJ21**	28.71k	4.33l	157.47e	5.17f	15.47d	4.29j
**GJ25**	30.62f	4.55i	170.59c	5.07j	13.89h	4.72h
**GJ3**	32.19c	5.48a	163.30d	5.22d	15.47d	4.98g
**GJ30**	29.02j	4.45j	176.01b	5.33a	14.89e	7.89a
**GJ5**	29.91g	4.90e	155.96e	5.22d	14.46f	4.68h
**GJ8**	27.70n	4.92e	168.83c	5.20e	16.36c	5.75e
**L1**	29.25i	4.61h	152.25f	5.29b	14.63e	5.16g
**LB10**	31.62d	4.71g	161.14d	5.23d	15.49d	4.91h
**LB101**	30.28g	4.78f	164.29d	5.21d	14.52f	3.98k
**LB15**	30.30g	4.94e	156.79e	5.20e	14.24g	4.41i
**LB30**	30.47f	4.82f	154.17f	5.15g	14.94e	5.04g
**LB33**	30.57f	4.32l	169.15c	5.13h	14.72e	4.98g
**LB68**	29.14j	4.64h	162.40d	5.15g	14.26g	5.07g
**LB80**	29.25i	4.32l	154.21f	5.15g	16.47c	5.65e
**LB88**	30.11g	5.00d	161.24d	5.26c	15.68d	6.23d
**N1**	30.09g	5.10c	165.44d	5.16f	13.33i	5.71e
**N13**	28.94j	4.94e	157.33e	5.19e	15.63d	5.11g
**N16**	30.99e	4.92e	167.50c	5.18e	14.60e	5.19g
**N2**	27.66n	4.33l	143.03h	5.22d	13.69h	5.47f
**N8(G8)**	27.96m	4.79f	167.58c	5.19e	15.31d	3.93k
**P42**	27.85m	4.66h	151.32f	5.11i	14.80e	5.45f
**P50**	28.35l	5.05c	168.17c	5.21d	16.47c	4.15j
**R152**	29.82g	4.79f	149.33g	5.23d	14.98e	4.64h
**R22**	28.92j	4.40k	149.44g	5.26c	14.55f	5.62e

**SK41**	28.58k	4.39k	155.02f	5.12h	14.01g	4.52i
**SK80**	28.24l	4.53i	144.65h	5.27b	13.87h	5.23g
**VP156**	30.46f	5.10c	170.71c	5.26c	14.68e	5.21g
**WP6**	29.68h	4.35l	154.50f	5.19e	14.56f	4.53i

d.b.: dry base. AE: aqueous extract (%d.b.), TA: total ash (%d.b.), TTA: total titratable acidity (mL NaOH 0.1 mol.L.100g^{-1} d.b.), pH: hydrogen potential (d.b.), TCP: total crude protein (%d.b.), EE: ether extract (%d.b.),

Supplementary material. Average grouping, according to Scott Knott at 5% probability, referring to the physicochemical characteristics of the most cultivated genotypes in the Western Amazon, Brazil, evaluated in two measurements, the 2020-2021 and 2021-2022 harvests, part 2.

Genótipos	TSS	ratio	TPC	SS	TRS	NRS
**31-131**	31.26c	0.19f	5.25g	8.17d	1.59d	6.58c
**AR106**	34.34a	0.21d	5.11h	8.92b	1.29h	7.63a
**AS1**	34.14a	0.20e	5.74b	8.49c	1.74b	6.75c
**AS10**	30.85c	0.19f	5.43e	3.66i	1.42f	2.24h
**AS12**	34.40a	0.20e	5.33f	7.22e	1.25i	5.96d
**AS2**	32.93b	0.20e	5.11h	8.97b	1.56d	7.41b
**AS3**	33.87a	0.19f	5.33f	9.58a	1.40g	7.85a
**AS5**	33.11b	0.20e	4.89j	8.35c	1.29h	6.73c
**AS6**	32.66b	0.18g	5.47d	7.52e	1.50e	6.02d
**AS7**	33.00b	0.20e	5.26g	9.02b	1.80b	7.68a
**BAG19**	30.91c	0.21e	4.45n	9.32a	1.30h	8.01a
**BAG21**	31.48c	0.23c	4.29o	7.96d	1.26i	6.70c
**BAG22**	34.09a	0.20e	5.68c	7.88d	1.56d	6.33c
**BAG23**	32.24b	0.22d	4.39n	5.46h	1.34h	4.12g
**BAG24**	32.03c	0.21d	4.79k	6.49g	1.15j	5.34e
**BAG26**	34.17a	0.22d	5.09h	7.01f	1.56d	5.45e
**BAG27**	33.00b	0.20e	5.33f	7.83d	1.53e	6.30c
**BAG28**	31.71c	0.21e	5.43e	8.64b	0.86l	8.05a
**BAG29**	33.86a	0.25a	5.27g	7.42e	1.38g	6.04d
**BAG30**	32.94b	0.22d	4.46n	8.60c	1.28h	7.32b
**BAG32**	32.05c	0.20e	5.14h	6.70f	1.43f	5.27e
**BAG33**	33.04b	0.23b	4.44n	7.60e	1.26i	6.35c
**BAG38**	34.16a	0.20e	5.39e	7.83d	1.46f	6.37c
**BAG41**	32.30b	0.23c	4.71l	8.68b	1.45f	7.24b

**BG180**	32.57b	0.20e	4.64m	7.32e	1.34h	5.98d
**BRS1216**	33.25b	0.20e	4.66m	7.27e	1.27i	6.00d
**BRS2299**	35.88a	0.21e	5.54d	7.78d	1.48e	6.30c
**BRS2314**	33.48b	0.23c	4.89j	8.47c	1.51e	6.96b
**BRS2336**	31.61c	0.20e	4.74l	8.10d	1.10j	6.99b
**BRS2357**	34.92a	0.21d	5.39e	7.09e	1.66c	5.43e
**BRS3137**	31.11c	0.20f	5.26g	6.95f	1.43f	5.52e
**BRS3193**	33.97a	0.19f	5.86a	9.84a	1.48f	8.03a
**BRS3210**	33.54b	0.21d	4.63m	7.87d	1.40g	6.47c
**BRS3213**	34.15a	0.22c	5.09h	8.13d	1.58d	6.55c
**BRS3220**	33.01b	0.21d	4.85k	7.29e	1.39g	5.89d
**CA1**	33.29b	0.18g	5.47d	7.43e	1.45f	6.01d
**GB1**	33.69a	0.21d	4.20p	7.37e	1.51e	5.86d
**GB4**	31.18c	0.20e	4.72l	7.67d	1.30h	6.37c
**GB7**	33.07b	0.21d	4.63m	5.13h	1.14j	3.99g
**GJ20**	30.63c	0.21e	4.21p	6.05g	0.90l	5.01f
**GJ21**	34.72a	0.22c	4.92j	7.51e	1.20i	6.32c
**GJ25**	33.95a	0.20e	5.11h	9.40a	1.43f	7.97a
**GJ3**	34.61a	0.24b	5.21g	5.03h	1.32h	3.71g
**GJ30**	32.57b	0.19g	5.27g	8.31c	1.39g	6.97b
**GJ5**	33.26b	0.21d	5.12h	8.43c	1.36h	7.33b
**GJ8**	33.33b	0.20e	5.64c	5.49h	1.98a	4.04g
**L1**	31.65c	0.21d	5.29f	7.80d	1.32h	6.49c
**LB10**	34.19a	0.22d	5.41e	5.32h	1.13j	4.19g
**LB101**	30.27d	0.19f	5.42e	8.66b	1.17j	7.47b
**LB15**	33.16b	0.21d	5.08h	8.80b	1.65c	7.16b
**LB30**	32.73b	0.21d	5.42e	9.39a	1.32h	7.99a
**LB33**	29.87d	0.18g	5.07h	5.89g	1.14j	4.67f
**LB68**	28.97d	0.18g	5.15h	8.60c	1.21i	7.22b
**LB80**	34.66a	0.23c	5.54d	5.02h	1.36h	3.65g
**LB88**	33.35b	0.21d	4.65m	8.86b	1.74b	7.28b
**N1**	32.66b	0.20e	4.80k	7.54e	1.33h	6.21d
**N13**	33.31b	0.21d	5.15h	7.81d	1.38g	6.43c
**N16**	32.56b	0.20f	5.76b	7.39e	1.76b	5.63e

**N2**	31.75c	0.22c	4.22p	6.25g	1.15j	5.10f
**N8(G8)**	32.84b	0.20e	4.98i	7.66d	1.38g	6.28c
**P42**	31.74c	0.21d	4.48n	7.42e	1.32h	6.09d
**P50**	32.40b	0.19f	5.20g	8.23c	1.56d	6.51c
**R152**	32.67b	0.22c	5.59c	6.44g	1.53e	4.92f
**R22**	33.14b	0.22c	4.70l	6.40g	1.09j	5.31e
**SK41**	32.38b	0.21d	4.55m	6.51g	1.11j	5.41e
**SK80**	29.05d	0.24b	4.66m	8.36c	1.17j	7.19b
**VP156**	29.64d	0.18g	5.23g	6.71f	1.10j	5.77d
**WP6**	34.04a	0.22c	4.31o	6.18g	1.00k	5.18f

d.b.: dry base. TSS: total soluble solids (%d.b.), ratio (% total soluble solids/mL of NaOH d.b.), TPC: total phenolic compounds (g gallic acid eq./100g of the sample d.b.), SS: total soluble sugars (% d.b.), TRS: total reducing sugars (%d.b.), NRS: non-reducing sugars (%d.b.).
